# IL-2 Immunotherapy Reveals Potential for Innate Beta Cell Regeneration in the Non-Obese Diabetic Mouse Model of Autoimmune Diabetes

**DOI:** 10.1371/journal.pone.0078483

**Published:** 2013-10-24

**Authors:** Yaiza Diaz-de-Durana, Janet Lau, Deborah Knee, Christophe Filippi, Marco Londei, Peter McNamara, Marc Nasoff, Michael DiDonato, Richard Glynne, Ann E. Herman

**Affiliations:** 1 Genetics Department, Genomics Institute of the Novartis Research Foundation, San Diego, California, United States of America; 2 Biotherapeutics Department, Genomics Institute of the Novartis Research Foundation, San Diego, California, United States of America; 3 Translational Medicine, Novartis Institutes of Biomedical Research, San Diego, California, United States of America; 4 Pharmacology Department, Genomics Institute of the Novartis Research Foundation, San Diego, California, United States of America; 5 Structural Biology Department, Genomics Institute of the Novartis Research Foundation, San Diego, California, United States of America; San Raffaele Scientific Institute, Italy

## Abstract

Type-1 diabetes (T1D) is an autoimmune disease targeting insulin-producing beta cells, resulting in dependence on exogenous insulin. To date, significant efforts have been invested to develop immune-modulatory therapies for T1D treatment. Previously, IL-2 immunotherapy was demonstrated to prevent and reverse T1D at onset in the non-obese diabetic (NOD) mouse model, revealing potential as a therapy in early disease stage in humans. In the NOD model, IL-2 deficiency contributes to a loss of regulatory T cell function. This deficiency can be augmented with IL-2 or antibody bound to IL-2 (Ab/IL-2) therapy, resulting in regulatory T cell expansion and potentiation. However, an understanding of the mechanism by which reconstituted regulatory T cell function allows for reversal of diabetes after onset is not clearly understood. Here, we describe that Ab/IL-2 immunotherapy treatment, given at the time of diabetes onset in NOD mice, not only correlated with reversal of diabetes and expansion of Treg cells, but also demonstrated the ability to significantly increase beta cell proliferation. Proliferation appeared specific to Ab/IL-2 immunotherapy, as anti-CD3 therapy did not have a similar effect. Furthermore, to assess the effect of Ab/IL-2 immunotherapy well after the development of diabetes, we tested the effect of delaying treatment for 4 weeks after diabetes onset, when beta cells were virtually absent. At this late stage after diabetes onset, Ab/IL-2 treatment was not sufficient to reverse hyperglycemia. However, it did promote survival in the absence of exogenous insulin. Proliferation of beta cells could not account for this improvement as few beta cells remained. Rather, abnormal insulin and glucagon dual-expressing cells were the only insulin-expressing cells observed in islets from mice with established disease. Thus, these data suggest that in diabetic NOD mice, beta cells have an innate capacity for regeneration both early and late in disease, which is revealed through IL-2 immunotherapy.

## Introduction

Type-1 diabetes (T1D) is an autoimmune disease targeting insulin-producing beta cells, resulting in a lack of insulin production that requires lifelong exogenous insulin administration. T1D presents predominantly in children, but can affect adults, and is associated with significant morbidity and mortality [1]. From a health economics and outcomes perspective, T1D accounts for approximately $12 billion USD annually in health care costs [2]. Non-obese diabetic (NOD) mice serve as a model for human T1D, and have proven to be a valuable tool to identify potential modes of disease pathogenesis and genetic susceptibility translatable to humans. Studies have implicated aberrant IL-2/IL-2 receptor (CD25) signaling as a contributing factor to both mouse and human T1D [3]. IL-2 is a cytokine important in the survival and function of CD4+CD25+ regulatory T cells (Treg) [4]. Indeed, a genetic predisposition to IL-2 deficiency in autoimmune diabetes impacts function of Treg in humans with T1D, as well as in NOD mice [5-9]. Moreover, studies have shown that treatment with exogenous IL-2 or Ab/IL-2 can selectively expand Tregs, improve immune regulation, and prevent or reverse disease at the onset of diabetes in NOD mice [5,6,10,11]. Based on these data, the use of low-dose IL-2 immunotherapy in T1D is under clinical investigation [12,13]. 

Functionally, Tregs have been reported to mediate IL-2 immunoregulatory effects [5,6]. However, the relationship between improved immunoregulation and reversal of hyperglycemia after diabetes onset remains unclear. Immunoregulation is thought to relieve T cell pressure on beta cells, thus preserving remaining beta cells, and allowing re-granulation and improved insulin secretion [1,14,15]. However, this mechanism alone may not fully explain a long-lasting return to normoglycemia, suggesting additional mechanisms are contributing to diabetes reversal. Recent studies using genetic or chemical ablation of beta cells in mice, suggest that beta cells may be able to regenerate through processes of proliferation, activation of progenitors, or alpha to beta cell transdifferentiation [16-20]. Regenerative processes have not been assessed as a mechanism for amelioration of diabetes in NOD mice with IL-2 immunotherapy. Here, we asked whether Ab/IL-2 immunotherapy in diabetic NOD mice could reveal an innate potential for beta cell regeneration. 

## Results

**Figure 1:**
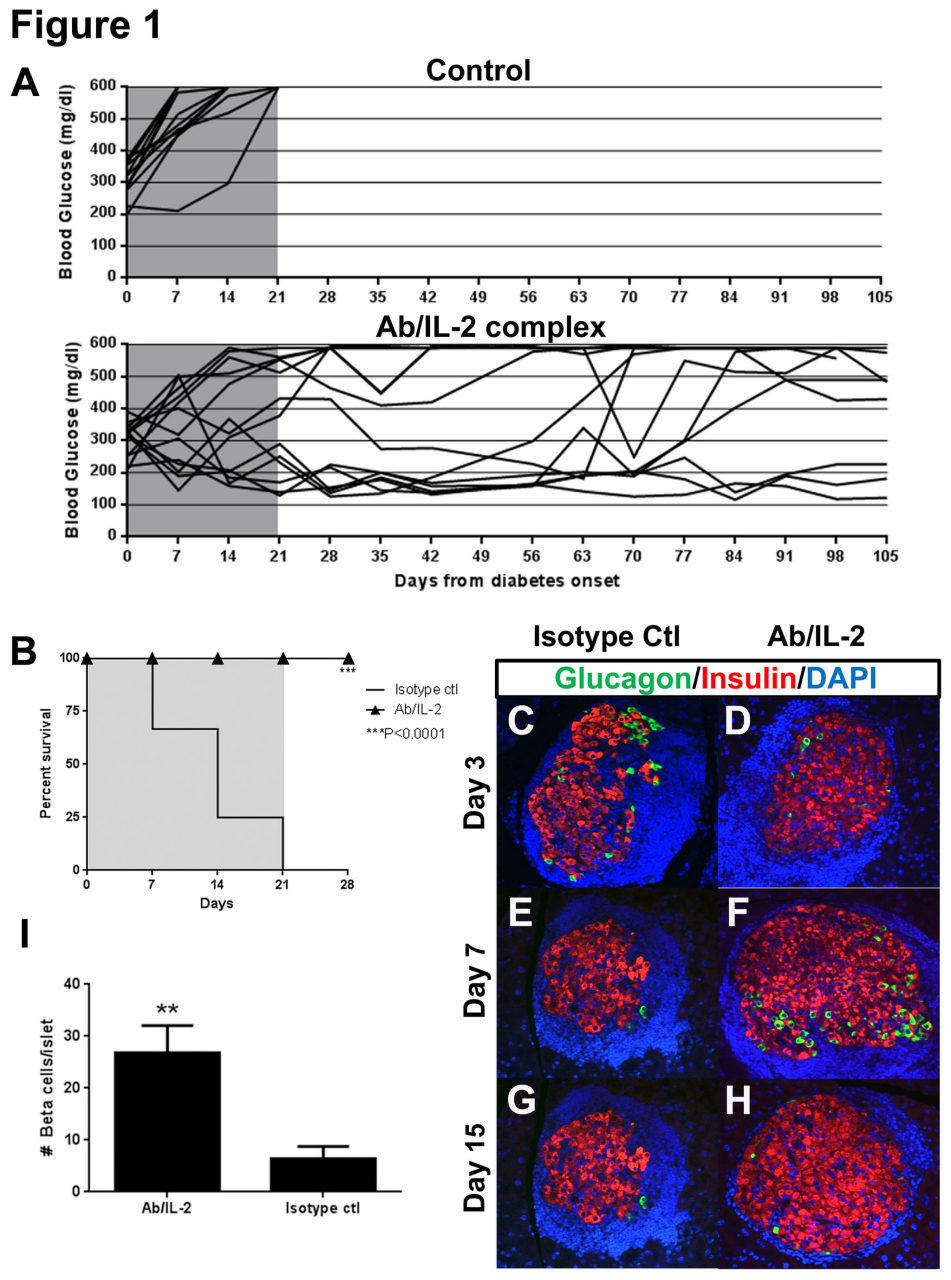
Ab/IL-2 immunotherapy enhances survival and preserves beta cells in diabetic NOD mice. NOD mice were diagnosed with recent-onset diabetes after two consecutive days with measurements of blood glucose between 200-400mg/dl. Diabetic mice were treated daily with intra-peritoneal injections of either control (PBS or isotype Ab) or Ab/IL-2 for 21 days (grey shaded area). Blood glucose (**A**), and survival (**B**) were monitored. (n=12 for isotype control; n=13 for Ab/IL-2). **C**-**H**. Pancreata from mice of Ab/IL-2 and control groups were processed for immunofluorescence staining for insulin (red), glucagon (green), and DAPI (blue) at the indicated number of days post-onset. **I**. Islets from Ab/IL2 treated mice retained a higher absolute number of insulin+ cells/islet compared with controls. Insulin+ beta cells in recently diabetic NOD pancreata were counted from n=3-4 mice per treatment group, from each of two representative non-consecutive sections per pancreas, with a minimum of 30 islets and 900 beta cells/group analyzed. Mean insulin+ beta cell number per islet ± standard deviation between individual mice is presented. (**P<0.009, ***P<0.0001) .

Previous studies demonstrated that Ab/IL-2 immunotherapy promoted selective expansion of Treg cells over auto-reactive effector T cells in NOD mice [6,10]. In order to test whether Ab/IL-2 immunotherapy could reveal innate regenerative processes in autoimmune diabetes, we treated recently diabetic NOD mice with Ab/IL-2 or control Ab daily for 3 weeks starting at disease onset. As previously reported [5,6], we observed an expansion of the CD4+CD25+FoxP3+ Treg cell populations in spleen, pancreatic lymph nodes, and pancreas-infiltrating lymphocytes of Ab/IL-2 treated mice compared with controls (Figure S1). Within 3 weeks of treatment, six out of thirteen diabetic NOD mice treated with Ab/IL-2 returned to normoglycemia; while twelve out of twelve control-treated diabetic mice became morbidly hyperglycemic (Figure 1a). Moreover, all diabetic NOD mice treated with Ab/IL-2 immunotherapy survived in the absence of insulin therapy, including those that remained hyperglycemic, whereas no control treated animals survived beyond 4 weeks (Figure 1b, ***P<0.0001). Interestingly, the 3-week course of Ab/IL-2 dosing was sufficient to restore long-term normoglycemia, or promote long-term survival in hyperglycemic mice, for more than 98 days without the need for exogenous insulin (Figure 1a). At onset of hyperglycemia, recently diabetic mice retain some beta cells; however, these cells are lost over time. To determine if Ab/IL-2 treated mice continue to experience progressive beta cell loss, islets from mice treated with either Ab/IL-2 or control Ab were assessed by histology over a time course after disease onset (Figure 1c-h, Figure S2). Three days after onset of hyperglycemia, the numbers and size of islets appeared similar between controls and Ab/IL-2-treated mice (Figure 1c, d). Between days 3 and 15 post-onset in control animals, beta cells were progressively lost (Figure 1c, e, g). In contrast, Ab/IL-2 treated mice did not demonstrate comparable beta cell loss (Figure 1d, f, h). To quantitate beta cell loss in islets, we counted all remaining insulin expressing beta cells in representative sections of total pancreata from each diabetic mouse. Numbers were compared between individual pancreata from mice treated with either Ab/IL-2 or control (Figure 1i). After 1 week of treatment, the absolute number of insulin+ beta cells per islet was significantly higher in pancreata from Ab/IL-2 treated mice than those from control-treated mice, corresponding to larger islets (Figure 1d, f, h, i, **P<0.009). These data indicate that indeed, Ab/IL-2 immunotherapy slowed beta cell loss when given at diabetes onset. 

**Figure 2:**
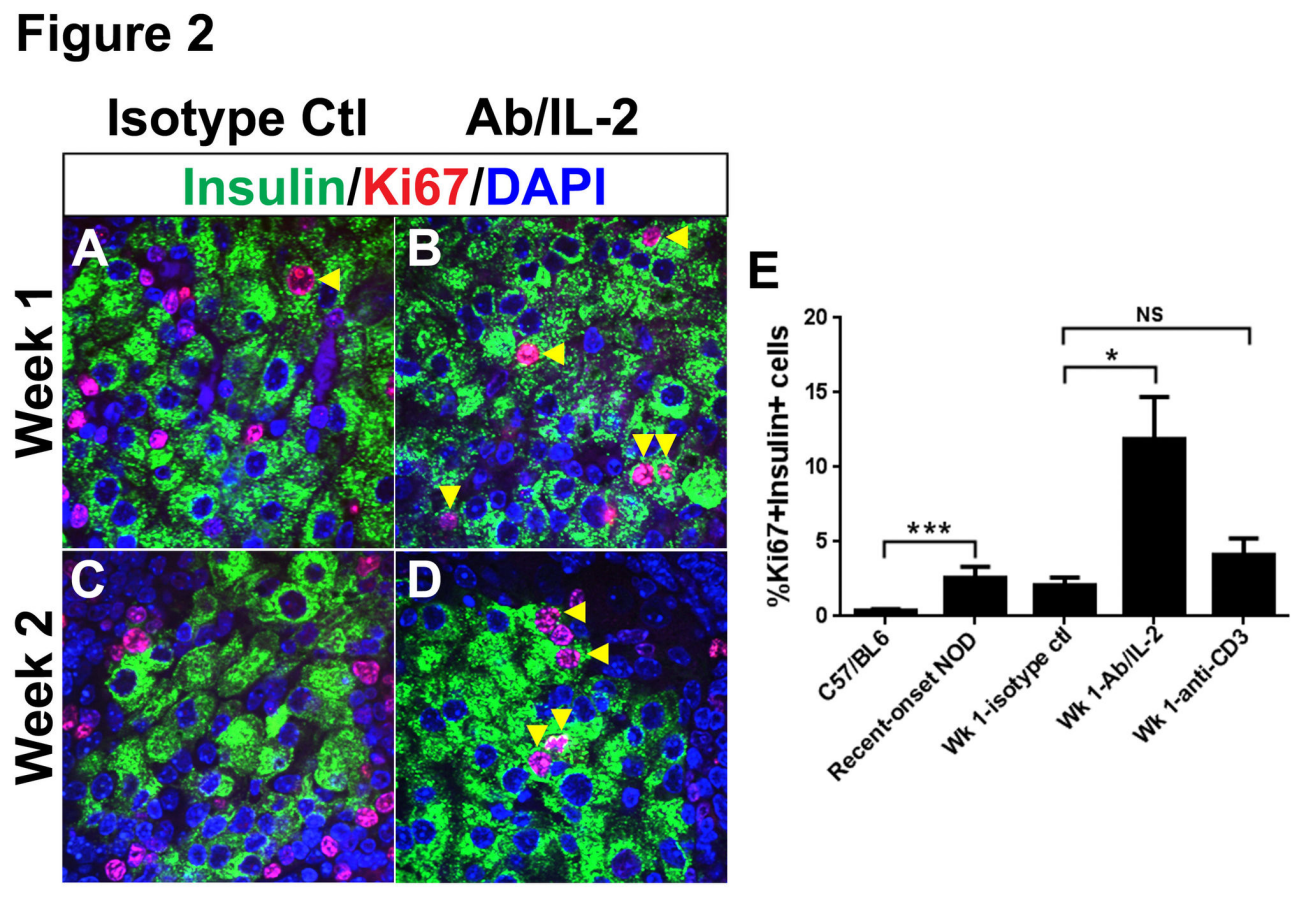
Ab/IL-2 immunotherapy augments regeneration through beta cell proliferation. **A**-**D**. Recently diabetic NOD mice were treated with Ab/IL-2 or control isotype Ab for one to two weeks, and pancreata were processed for immunofluorescence staining for insulin (green), Ki67 (red), and DAPI (blue). **E**. Mean percent of proliferating Ki67+/insulin+ beta cells of total insulin+ beta cells was calculated from mouse pancreata samples collected during the first week of treatment by counting beta cells (blinded to treatment group) from n=4-8 mice per group, from each of two representative non-consecutive sections per pancreas, with a minimum of 1800 insulin+ cells per group counted + standard error of the mean. (*P<0.013, *** P<0.0008).

Next, we assessed whether simple preservation of existing cells or regeneration might account for decreased beta cell loss after Ab/IL-2 immunotherapy. Previous studies have demonstrated that NOD beta cells have a high rate of proliferation at diabetes onset compared with non-autoimmune strains [14,21]. Since the retention of beta cells with Ab/IL-2 immunotherapy could reflect either preservation of existing mass and/or replication, we histologically assessed proliferation in islets from recently diabetic NOD mice (Figure 2, Figures S3, S4). Control or Ab/IL-2 treated pancreata were processed for immunofluorescence staining with insulin and Ki67, within the first or second week after diabetes onset (Figure 2a-d, Figures S3, S4). The percentage of insulin+ beta cells expressing Ki67 was quantitated (Figure 2e). As reported [14,21,22], beta cells from recent-onset diabetic NOD mice demonstrated a burst of proliferation in comparison to C57BL6 mice (Figure 2e, ***P<0.0008). No difference was observed between untreated recent-onset diabetic mice compared with control isotype Ab treated mice (Figure 2e). Interestingly, beta cells from Ab/IL-2 treated mice showed a robust and significantly enhanced proliferation frequency, compared with beta cells from control-treated diabetic NOD in the first week after onset (Figure 2e, *P<0.013). We note that in a previous study, a decrease in beta cell proliferation was seen after anti-CD3 immunotherapy when assessed at 3 weeks post-diabetes onset [14]. Therefore, we directly compared anti-CD3 with Ab/IL-2 immunotherapy for effects on beta cell proliferation after one week of treatment (Figure 2e). As previously published [23-26], anti-CD3 therapy was effective at restoring normoglycemia in the majority of diabetic mice at disease onset (data not shown). However, beta cells from anti-CD3 treated diabetic mice did not proliferate significantly more than control-treated mice, in contrast to beta cells from Ab/IL-2 treated mice (Figure 2e). Based on this comparison, immunomodulation with Ab/IL-2 demonstrated an improved effect on regeneration through proliferation, compared with mouse anti-CD3 therapy. Anti-CD3 has been documented to enhance the frequency of Treg cells [26-28], similar to Ab/IL-2 therapy. However, IL-2 may also improve immunoregulation by directly enhancing Treg function, potentially encouraging improved tissue repair. Proliferation in islets from Ab/IL-2 treated mice correlated with the timing of significant islet size reduction and loss of beta cells in control NOD mice, suggesting that proliferation is contributing to maintenance of beta cell numbers in Ab/IL-2 treated mice. Thus, Ab/IL-2 immunotherapy promotes immunoregulation when given at disease onset, and also allows for significant enhancement of regeneration predominantly via proliferation.

**Figure 3:**
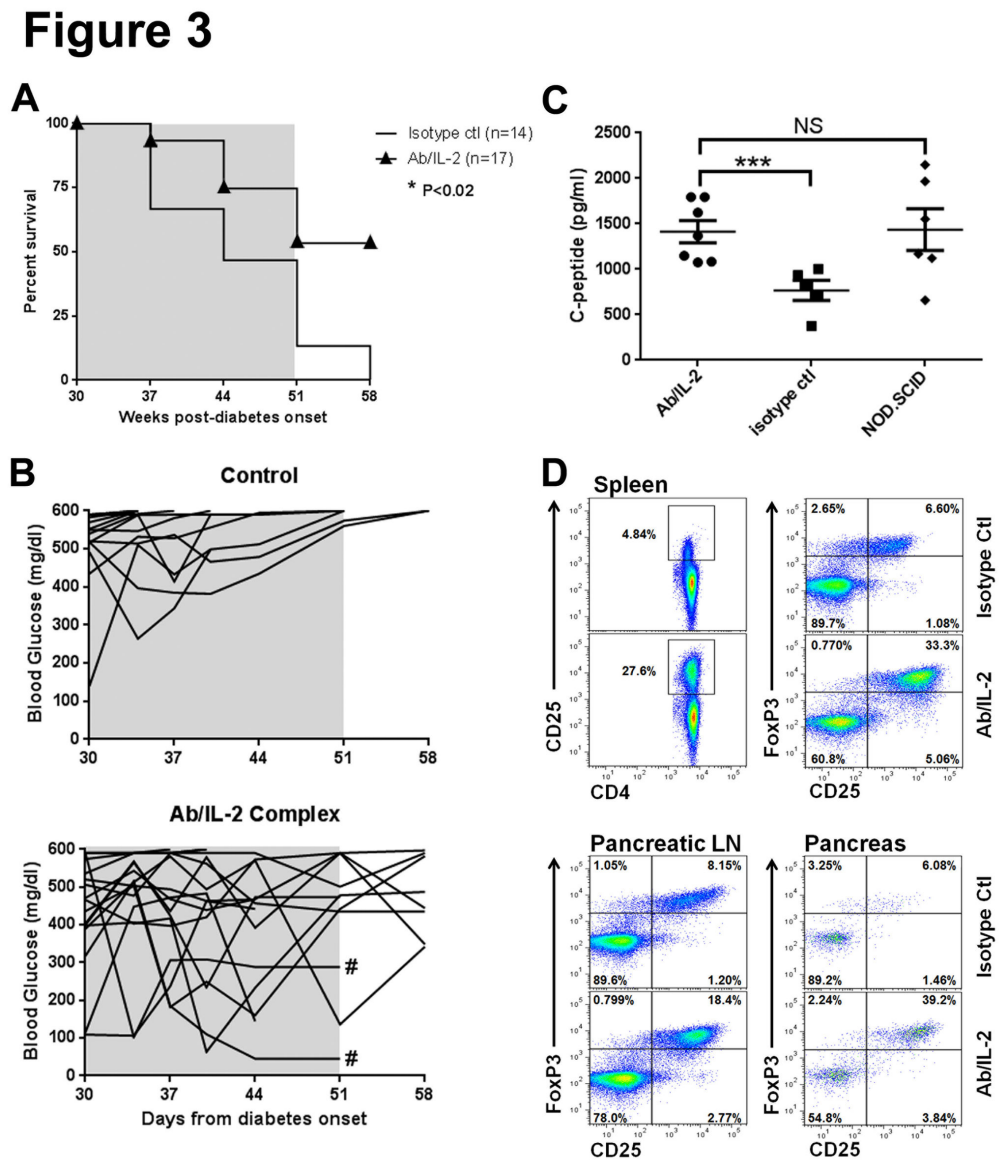
Ab/IL-2 immunotherapy promotes survival and C-peptide production in established diabetic NOD mice. Mice diagnosed with fulminant diabetes at onset (>400mg/dl) were treated with insulin pellets to sustain survival. Starting 4 weeks after diabetes onset, mice were treated with Ab/IL-2 or control isotype Ab for 21 days (from week 4-7 post-onset, grey shaded areas), and further insulin therapy was withheld. Survival (**A**) and blood glucose (**B**) were monitored (n=14 isotype control, n=17 Ab/IL-2 treated). **C**. C-peptide levels were measured in established diabetic mice treated with Ab/IL-2 or control Ab from week 4-7 after onset. Levels after Ab/IL-2 treatment improved and were similar to age-matched control non-diabetic NOD.SCID mice. **D**. Established diabetic mice were treated for 7 days with Ab/IL-2 or isotype control Ab. Splenocytes, pancreatic lymph node (LN) and pancreatic lymphocytes from n=3 pooled treated mice for each group were analyzed by flow cytometry as described in methods. Plots shown are gated on CD4+ T cells. Treg cells were selectively expanded by Ab/IL-2 treatment in established diabetic NOD mice, as evidenced by increased % of CD4+CD25+FoxP3+ cells. (***P<0.0003, **#**-Mice sacrificed for histology analysis.) .

Given that immunoregulation with Ab/IL-2 immunotherapy was able to promote beta cell regeneration at disease onset, we postulated that regeneration might be operable even at later stages of disease. Therefore, we developed a model of long-term diabetes to assess whether Ab/IL-2 immunotherapy could still have an impact via regeneration. NOD mice with severe diabetes at onset (blood glucose >400mg/dl), were treated with insulin for 4 weeks after the initial onset of disease. These mice are further referred to as “established” diabetic mice. They were then treated daily with Ab/IL-2 or control therapy daily for the following 3 weeks (from 4 to 7 weeks after initial disease onset), with no further exogenous insulin added (Figure 3). Beta cells were rare or absent in the pancreata of NOD mice with established diabetes of this duration. However, 50% of animals treated with Ab/IL-2 immunotherapy survived without further insulin therapy (Figure 3a, *P<0.02), and a few demonstrated improved glycemia (Figure 3b). Similar to recent-onset diabetic NOD mice, control animals with established diabetes succumbed to disease within approximately 3 weeks of the end of insulin therapy (Figure 3b). In addition, we found that insulin production was partially restored, as demonstrated by improved C-peptide levels in established diabetic mice treated with Ab/IL-2 compared with control treatment (Figure 3c, ***P<0.0003). While C-peptide levels appeared equivalent to levels in non-diabetic NOD.scid mice, clearly, the insulin produced was not sufficient to achieve normoglycemia in most animals. This suggests that the quality of insulin/C-peptide produced was not fully functional, and/or that other factors existed which prevented normoglycemia in established diabetic NOD mice. Indeed, extenuating factors which could explain this observation, including insulin resistance, impaired insulin processing, aberrantly folded insulin, or glucagon dysregulation, have been reported in diabetic NOD mice [29-33]. In order to assess whether Ab/IL-2 immunotherapy still expanded Tregs in NOD mice with established diabetes, we analyzed spleen, pancreatic lymph node, and pancreatic lymphocytes by flow cytometry (Figure 3d). As in recent onset diabetic mice treated with Ab/IL-2 treatment, CD4+CD25+FoxP3+ Tregs in established diabetic NOD mice were significantly expanded after 1 week of Ab/IL-2 treatment, confirming immunoregulation as a mechanism for Ab/IL-2 therapy at both stages (Figure 3d, Figure S1). These findings indicate that Ab/IL-2 induction of immunoregulation in established diabetes has beneficial effects by promoting insulin production and extending survival.

**Figure 4:**
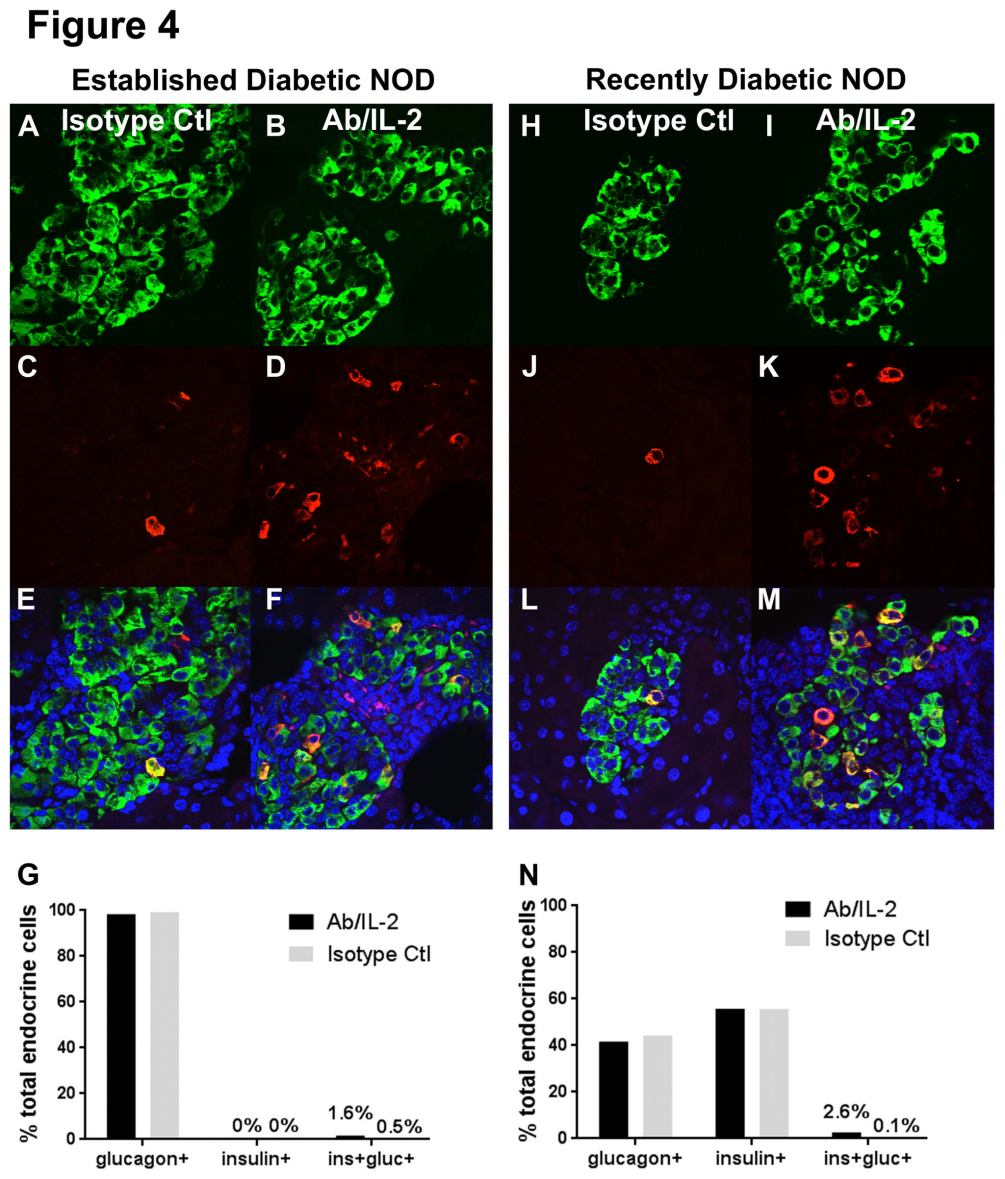
Ab/IL-2 immunotherapy enhances a rare dual-expressing insulin+/glucagon+ cell population in diabetic NOD mice. Established or recently diabetic NOD mice were treated with Ab/IL-2 or isotype control Ab for 3 weeks, as described. Pancreata were harvested and processed for immunofluorescence staining for insulin (red), glucagon (green), and DAPI (blue). Insulin+/glucagon+ cell numbers appeared significantly increased by Ab/IL-2 treatment in established diabetic (**A**-**G**) and recent onset diabetic (**H**-**N**) NOD pancreata. Notably, in established diabetic NOD mice, insulin+/glucagon+ cells were the only detectable source of insulin. (**G**, **N**) Percentage of insulin+, glucagon+, and dual-expressing insulin+/glucagon+ (yellow) cells were quantitated by counting all islet cells from each of two representative, non-consecutive sections per pancreas per group. **G**. In established diabetic mice, a total of n=2898 Ab/IL-2 islet cells and n=1498 control islet cells (n=4-8 mice per group) were counted; (**N**) in recent onset diabetic mice, n=2264 Ab/IL-2 islet cells and n=961 control islet cells (n=3-4 mice per group) were counted. Due to the rarity of islets themselves and insulin+/glucagon+ cells within the islet, cells from all pancreata within each group were pooled in order to determine the overall percentage. Thus, statistical comparison between individual animals could not be performed.

The improvement in C-peptide levels in established diabetic mice was surprising given that these mice had virtually no remaining normal insulin-expressing beta cells (Figure 4a-c). However, we observed a source of insulin expression in dual-expressing insulin+/glucagon+ cells in the pancreas, which appeared at higher frequency in Ab/IL-2 compared with control-treated mice (Figure 4a-f). To determine the representative percentage of insulin+/glucagon+ cells found in any remaining islets, we quantitated the frequency of insulin+/glucagon+ cells from pancreata of established diabetic mice treated with either Ab/IL-2 or control (Figure 4g). Although rare, these cells were increased in Ab/IL-2 compared with control-treated mice (Figure 4g, 1.6% vs. 0.5%). No other sources of insulin expression could be detected in other tissues such as gut or liver (unpublished observations). Notably, insulin+/glucagon+ cells in pancreata also co-expressed C-peptide (Figure S5). Therefore, these insulin+/glucagon+ cells appeared to be the source of C-peptide detected in Ab/IL-2 treated established diabetic mice (Figure 3c, Figure S5). Although small, the increased frequency of insulin+/glucagon+ cells and enhanced levels of C-peptide observed in Ab/IL-2 treated mice suggested that these cells may have achieved a functional threshold of insulin production that could explain their improved survival, when compared with control treated mice with established diabetes.

Notably, in recent-onset diabetic mice that retain significant numbers of insulin-expressing beta cells, rare insulin+/glucagon+ cells were also observed (Figure 4h-n). Again, these cells were more prevalent in Ab/IL-2 treated mice (Figure 4n; 2.6% vs. 0.1% in Ab/IL2 and control mice, respectively). Importantly, insulin+/glucagon+ cells were present, albeit at much lower frequency, in pancreata of control treated mice (Figure 4h, j, l, n). These data are consistent with beta cell regeneration in a process enhanced by Ab/IL-2 treatment. Furthermore, these data indicate that regenerative processes occur naturally in diabetic mice, and are enhanced by Ab/IL-2 treatment. Taken together, Ab/IL-2 immunotherapy may reveal regenerating beta cells otherwise suppressed or killed by autoimmunity. 

**Figure 5:**
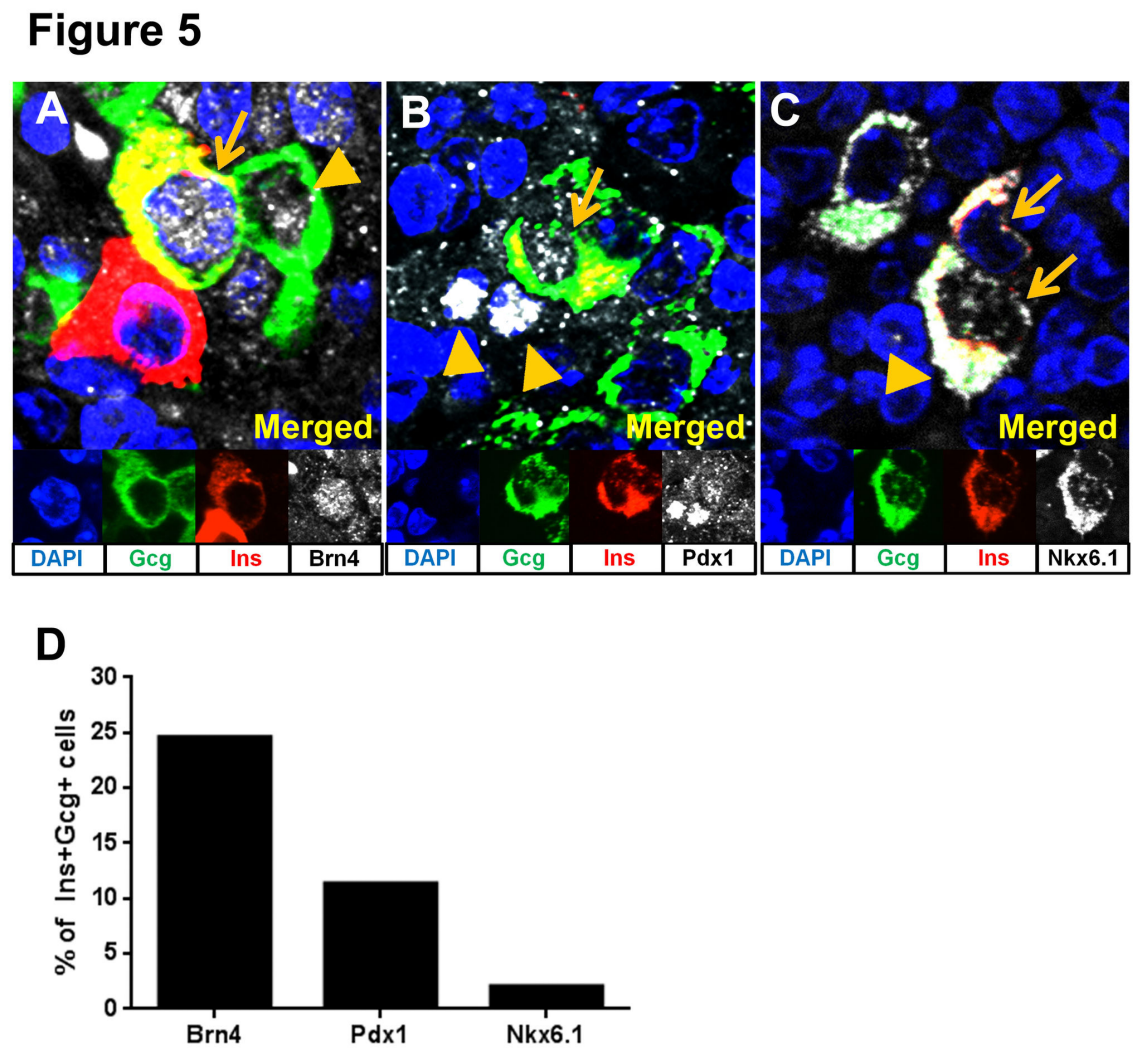
Insulin+/glucagon+ cells do not reflect mature endocrine cells. Recent onset diabetic NOD pancreata treated with Ab/IL-2 were processed and stained for insulin (red), glucagon (green), DAPI (blue) and mature endocrine markers, Brn4, Pdx1, or Nkx6.1 (white). (**A**) Staining for Brn4, a transcription factor expressed in mature alpha cells, showed expression in glucagon+ (arrowhead) and insulin+/glucagon+ (arrow) cells. (**B**) Staining for Pdx1, a beta cell specific transcription factor, showed weak expression in insulin+/glucagon+ (arrow) cells, as well some hormone negative cells (arrowhead). (**C**) Staining for Nkx6.1 showed only weak or absent nuclear staining (arrows) in insulin+/glucagon+ cells, as well as abnormal cytoplasmic staining (arrowhead). (**D**) Graph represents quantitation of the percent of insulin+/glucagon+ cells that also express nuclear Brn4, Pdx1 or Nkx6.1 (n=97+ insulin+/glucagon+ cells analyzed per transcription factor, from n≥4 individual samples).

Insulin+/glucagon+ cells are marked by abnormal expression of mature alpha and/or beta cell transcription factors in regeneration models [19,20]. However, this phenomenon has not been previously characterized in the NOD mouse. Therefore, we investigated expression of mature alpha and beta cell specific transcription factors in endocrine cells from pancreata of Ab/IL-2 treated NOD mice shortly after diabetes onset (Figure 5). Specifically, we examined Brain4/Pou3f4 (Brn4) expression as a marker of mature alpha cells, and Pdx1 and Nkx6.1 as markers of mature beta cells. Nkx6.1 expression has been previously described in insulin+/glucagon+ cells [20]. In pancreata from recently diabetic NOD mice treated with Ab/IL-2, nuclear expression of Brn4 was observed in ~25% of insulin+/glucagon+ cells (Figure 5a, d), while a smaller proportion expressed low-level nuclear Pdx1 (Figure 5b,d). Nuclear staining of Nkx6.1 was present weakly, if at all, in a minority of insulin+/glucagon+ cells, but was expressed in the cytoplasm of all insulin+/glucagon+ cells (Figure 5c, d). 

Interestingly, we also observed widespread, abnormal expression of both Pdx1 and Nkx6.1 transcription factors in the pancreata of recent-onset diabetic NOD mice, including Pdx1+ expression in 6% of alpha cells (Figure S6a-d, m), and cytoplasmic Nkx6.1 expression also in 94% of mature alpha cells and 1% of beta cells (Figure S6i-l, n). In addition, we identified 33% of islet cells as insulin-/glucagon-/Pdx1+ cells (Figure 5b, arrowheads, and Figure S6e-h, m), which may represent degranulated beta cells, as previously described [14]. No expression of Brn4 was detected in beta cells (data not shown). To confirm Ab specificity, transcription factor staining was verified in normal, corresponding mature alpha and beta cells from study samples (Figure 5a, arrowhead). Abnormal expression of Pdx1 and Nkx6.1 in endocrine cells may be indicative of an altered state of differentiation that is permissive for regeneration. Therefore, these data suggest that insulin+/glucagon+ cells are neither fully differentiated alpha nor beta cells, but rather intermediate endocrine cells capable of dual hormone expression. 

Taken together, examination of regeneration in diabetic NOD mice treated with Ab/IL-2 therapy suggests that the autoimmune-induced death of beta cells and/or the hyperglycemic state might be creating a stimulus for innate regeneration. Restoration of immune regulation through Ab/IL-2 immunotherapy enhanced regenerative processes through beta cell proliferation, de-regulated mature alpha and beta cell transcription factor expression, and induced abnormal insulin expression in pancreata of mice with recent-onset or established autoimmune diabetes.

## Discussion

In our study, enhanced beta cell proliferation was identified as a potential mechanism for amelioration of diabetes via restored immunoregulation in recent-onset diabetic NOD mice. Ab/IL-2 immunotherapy not only preserved beta cell numbers in recently diabetic mice, but also promoted proliferation, while anti-CD3 immunotherapy did not. Yet, agents that have promoted beta cell proliferation in rodent models have not translated into efficacy in human clinical trials. Moreover, several studies have suggested that human beta cells do not proliferate beyond early life [34-37]. This is in contrast to rodent beta cells that appear to retain a higher and longer capacity for proliferation during adulthood [16,37-39]. However, reports of naturally occurring beta cell proliferation in recent-onset T1D patients with insulitis (27), and in an individual that presented positive for three autoantibodies and insulitis (26), suggest that special circumstances may exist in autoimmunity that are permissive for human beta cell proliferation. Thus it may be feasible that IL-2 could restore immunoregulation and enhance the natural proliferation of human beta cells, alone, or in combination with small molecule therapy to directly induce human beta cell proliferation [40]. 

In addition to proliferation, we identified insulin+/glucagon+ endocrine cells in NOD mice treated with Ab/IL-2 immunotherapy as a potential source of insulin. Insulin+/glucagon+ cells have also been described as intermediaries in alpha-to-beta cell transdifferentiation [19,20]. In the absence of genetic lineage tracing constructs, we could not determine the origin or terminal fate of the dual-expressing cells in diabetic NOD mice. However, the emergence of insulin+/glucagon+ cells, along with abnormal expression of beta cell markers in alpha cells, could suggest an alpha to beta cell transition as a possible mechanism of regeneration. These observations would be in agreement with Bramswig et al. who demonstrated that there is a greater epigenetic plasticity for alpha cells to express beta cell genes than the converse [41]. While speculative, these data support the regenerative potential of alpha cells as a source of insulin, which may be increased by IL-2 immunotherapy. 

While findings with IL-2 immunotherapy in mice appear promising, a recent study suggests caution for the clinical use of IL-2 immunotherapy [12]. Long et al showed that IL-2/rapamycin combination therapy induced Treg expansion in T1D patients, but patients nevertheless demonstrated impaired beta cell function. Importantly, the dosing regimen led to an increase in activated NK cells, which may have contributed to reduced beta cell function. The IL-2 dose was also three-fold higher than that used in a successful clinical trial of IL-2 to expand Treg in HCV-induced vasculitis [42]. These findings underscore the importance of IL-2 dosing as a key issue for clinical translation [8,43-46]. In addition, rapamycin has been reported to have negative effects on beta cell function, including in conjunction with anti-CD3 immunotherapy [47,48]. While the direct cause of reduced beta cell function in the IL-2/rapamycin study remains unclear, it highlights the continued need to focus on optimizing regimens for safe, effective use of IL-2 immunotherapy in T1D patients.

Currently, even the most clinically successful immune interventions are most effective when given within 6 months of disease onset in human T1D [49]. Therefore, establishing an effective immunotherapy for treatment of long-term diabetic patients remains an even greater challenge. However, several reports indicate that pancreata from established T1D cadavers still contain insulin-expressing cells [50-52]. These residual cells may reflect small pools of regenerating cells that are continually suppressed by the immune system, possibly similar to those observed in established diabetic NOD mice. Here, we observed insulin expression in dual-expressing insulin+/glucagon+ cells along with widespread de-regulation of alpha and beta cell transcription factors. A key question is whether immunotherapy would be successful in permitting beta cell regeneration in established T1D patients. A controversial clinical study consistent with this possibility demonstrated modestly upregulated C-peptide production and reduced insulin usage in a subset of established T1D patients treated with rapamycin immunosuppression prior to transplantation [53]. Importantly, retention of even small amounts of C-peptide has been demonstrated to reduce the risk of hypoglycemic events, the most significant cause of mortality with insulin therapy, as well as reducing the incidence of complications of T1D [54,55]. More recently, Wang et al. were able to demonstrate beta cell regeneration using an induced mixed chimerism and gastrin/EGF combination therapy supporting the possibility for beta cell regeneration in established diabetic NOD mice [56]. Our findings demonstrate the ability to enhance insulin expression in the absence of remaining beta cells, using Ab/IL-2 immunotherapy in established T1D in NOD mice. Taken together, these data imply that ongoing auto-reactivity is suppressing innate regenerative potential that exists in the endocrine/beta cell compartment in NOD mice, and suggest the possibility that appropriate immune modulation may be harnessed to treat established diabetes.

## Materials and Methods

###  Mice

Female non-obese diabetic (NOD) mice were purchased from Jackson Labs (Bar Harbor, ME). Mice were obtained at 8 weeks, aged, and monitored for diabetes onset weekly by blood glucose. Mice were considered recent-onset diabetic when they reached fed blood glucose values between 200-400 mg/dl, in two consecutive daily measurements. Blood glucose was monitored using the OneTouch® glucometer and test strips (Lifescan, CA). Mice used in established disease experiments were identified at onset with higher hyperglycemic measurements of >400mg/dl, and confirmed diabetic upon two consecutive daily measurements. These severely hyperglycemic mice were implanted with one or two Linbit® insulin pellets, based on the manufacturer’s instructions (LinShin, Canada). Insulin pellets dissolve within 4-6 weeks and were used to keep mice alive for this time frame, but no additional effort was made to closely control glucose levels. Four weeks after diagnosis and start of insulin treatment, established diabetic mice were enrolled in experiments. All animal experiments were performed in accordance with IACUC guidelines (protocol 11-291). 

### Immunotherapy

Recent-onset or established diabetic mice were treated with Ab/IL-2 comprised of 50 μg clone JES6-1A12 Rat IgG2a Ab (Bio X Cell, West Lebanon, NH) pre-mixed for 15-30 minutes before injection with 1.5 μg of recombinant mouse IL-2 (Ebioscience, San Diego, CA) in PBS. Ab/IL-2 complexes were injected intraperitoneally (i.p.) daily for 21 days, unless otherwise noted. Control treatment consisted of same volume PBS or where indicated 50 μg isotype-control Ab clone 2A3 Rat IgG2a Ab (Bio X Cell, West Lebanon, NH) in PBS. For anti-CD3 studies, anti-CD3 treatments were dosed intravenously (i.v.) with 10ug/mouse/day for five consecutive days using purified hamster anti-mouse CD3 mAb (BD Pharmingen #553058, clone 145-2C11) in PBS. Mice were monitored for blood glucose as above. 

### Immunohistochemistry and imaging

Pancreata were isolated into 10% neutral buffered formalin, washed with PBS, and stored in 70% ethanol until processing and embedding. Standard methods were used to obtain paraffin sections. 5μ sections were stained with Guinea Pig anti-insulin (DAKO, #A0654), Rabbit anti-insulin (Santa Cruz, #SC-9168), Rabbit anti-glucagon (DAKO, #A0565), Goat anti-glucagon (Santa Cruz, #SC-7780), Mouse anti-glucagon (Sigma, #G2654), Rabbit anti-C-peptide (Abcam, #AB14181), Mouse anti-Ki-67 (Affinity, #MAL-25918), Rabbit anti-PDX1 (Abcam, #AB47267), Mouse anti-Nkx6.1 (Dev. Studies Hybridoma Bank, #F55A10), Rabbit anti-Brn4/Pou3F4 (Sigma, #HPA031984), and/or DAPI. Non-consecutive sections were obtained at 50μ distance to ensure analysis of distinct islets, and were selected to be representative of typical pancreatic sections. Sections were analyzed using an Olympus Ultraview Confocal Microscope System. Cell count analysis was conducted visually over all available islets across two non-consecutive sections, per animal, per group, with n’s as reported in legends. In established diabetic mice sacrificed after treatment 7-11 weeks post diabetes onset, 7/8 mice had no conventional insulin+ beta cells upon sectioning through entire pancreata. 1 islet containing ~10 beta cells was identified in the 8^th^ mouse. For insulin+/glucagon+ cell counts in established diabetic mice, cell counts from pancreata of n=3-8 mice per treatment group were pooled in order to have enough cells for quantitation due to the rarity of islets and cells in these pancreata, therefore comparative statistics of individual animals were not performed. 

### FACS analysis

Tissue collection and processing of spleens, pancreatic lymph nodes and pancreatic lymphocytes were performed in RPMI (10%FBS) on ice. Tissues were processed with a syringe plunger through a 100μ screen to obtain single cell suspensions, and washed in RPMI (10% FBS). Red blood cells were lysed with Red Blood Cell Lysis Buffer (Sigma #R7757). FACS staining was performed under standard protocols using FACS buffer (1xPBS + 0.5% BSA + 0.05% sodium azide). Cells were stained with surface antibodies: Rat anti-mouse CD4-Pacific Blue (BD Pharmingen #558107), Rat anti-mouse CD8-PerCp (BD Pharmingen #553036), and Rat anti-mouse CD25-APC (Ebioscience #17-0251), and subsequently fixed/permerabilized and stained for FoxP3 according to the Anti-Mouse/Rat FoxP3 Staining Set PE (Ebioscience #72-5775). Cells were analyzed on the BD LSR Fortessa or BD FACS LSR II, and data analyzed with FlowJo software (TreeStar). 

### C-peptide analysis

Serum samples from established diabetic mice treated with either Ab/IL-2 or isotype control Ab from week 4-7 after diabetes onset, were collected at time points of ≥11 weeks after diabetes onset, or samples were collected from age-matched female NOD.scid mice, which share the NOD genetic background, but do not develop diabetes. Serum samples were stored at -20C until assessment. Mouse C-peptide levels in sera were measured by Bachem C-Peptide (mouse)-ELISA kit (#S-9104) according to the manufacturer’s protocol. The manufacturer certifies the ELISA kit reagents to have <5% cross-reactivity to either mouse insulin or proinsulin. 

## Supporting Information

Figure S1 **Ab/IL-2 immunotherapy expands Treg cells in recently diabetic NOD mice.** Mice recently diagnosed with diabetes were treated for 7 days with Ab/IL-2 or isotype control Ab. Splenocytes, pancreatic lymph node (LN) and pancreatic lymphocytes from n=3 pooled treated mice for each group were analyzed by flow cytometry, as described in methods. Plots shown are gated on CD4+ T cells. Treg cells were selectively expanded by Ab/IL-2 treatment in recently diabetic NOD mice, as previous published (Tang et al [6]) and evidenced here by increased % of CD4+CD25+FoxP3+ cells.
(TIF)

Figure S2 **Ab/IL-2 immunotherapy preserves beta cell area.** Wide-field images of insulin (red), glucagon (green) and DAPI (blue) stained pancreata presented at 5x magnification. (**A**, **D**) At day 3, an equivalent number of islets of comparable size was observed within all pancreata samples. (**B**, **E**) By day 7, a progressive loss of islet beta cells was apparent in isotype control treated samples, while Ab/IL-2 samples maintained significant beta cells in islets. (**C**, **F**) At day 15, isotype control treated islets were even more reduced in size and number, in comparison to Ab/IL-2 treated samples that had larger islets with more beta cells.
(TIF)

Figure S3 **Insulin and Ki67 staining in recently diabetic NOD mice treated with Ab/IL-2 immunotherapy.** Single channel and merged images of insulin (green), Ki67 (red) and DAPI (blue) staining in recent onset diabetic NOD mice treated for either one or two weeks with isotype control or Ab/IL-2 immunotherapy. (**A**-**D**, and **I**-**L**) Isotype control treated samples show few if any proliferating beta cells. (**E**-**H**, and **M**-**P**) In contrast, Ab/IL-2 treated samples show multiple proliferating beta cells after one or two weeks of treatment.
(TIF)

Figure S4 **Beta cells demonstrate increased proliferation with Ab/IL-2 immunotherapy.** Wide-field images of insulin (green), Ki67 (red) and DAPI (blue) stained pancreata presented at 5x magnification. After one or two weeks of immunotherapy, few Ki67+ beta cells were observed in isotype control treated samples (**A**, **B**), while a number of proliferating Ki67+ beta cells were observed in Ab/IL-2 treated samples (**C**, **D**).
(TIF)

Figure S5 **Insulin+/glucagon+ dual-expressing cells co-express C-peptide.** Rare insulin (red) and glucagon (green) dual-expressing cells also co-express cytoplasmic C-peptide (white). C-peptide co-expression was observed in recently diabetic NOD mice treated with either isotype control (**A**-**E**) or Ab/IL-2 (**F**-**J**) immunotherapy, or in established diabetic NOD mice also treated with either isotype control (**K**-**O**) or Ab/IL-2 (**P**-**T**) immunotherapy.
(TIF)

Figure S6 **Characterization of abnormal beta cell marker expression in Ab/IL-2 immunotherapy treated islets.** Recent-onset NOD mice were treated with Ab/IL-2 or control isotype Ab for 7 days. Pancreata were harvested, processed and stained for insulin (red), glucagon (green), DAPI (blue), and either Pdx1 or Nkx6.1 (white) antibodies. Insulin cells expressed nuclear Pdx1 (arrowhead) as expected (**A**-**D**), however occasional glucagon cells showed abnormal expression of nuclear Pdx1 (arrows) (**A**-**H**). Widespread hormone negative cells in the center of islets expressed nuclear Pdx1+ and may indicate degranulated beta cells (**E**-**H**). Insets show high magnification images of most Pdx1-/insulin+/glucagon+ cells (**E**’-**H**’). While insulin+ beta cells normally expressed nuclear Nkx6.1 (**I**’-**L**’), most hormone positive cells in diabetic NOD islets showed abnormal cytoplasmic Nkx6.1 expression (**I**-**L**), including insulin+/glucagon+ cells (arrowheads). **M**. Graph representing the percentage of abnormal Pdx1+ expressing cells, including the percent of glucagon+/Pdx1+ alpha cells, and percent of insulin-/glucagon-/Pdx1+ from total islet cell numbers (n=1730 total islet cells, including 943 alpha cells analyzed for Pdx1 expression from n=3 animals.) **N**. Graph representing the percentage of cells with cytoplasmic Nkx6.1 expression, including the percent of insulin+/Nkx6.1+ beta cells, and percent of glucagon+/Nkx6.1+ alpha cells (n=1514 total islet cells analyzed for cytoplasmic Nkx6.1+ cells, including 816 alpha cells and 158 beta cells from n=4 animals).
(TIF)
