# Supplementary figures and images for: IL-2 Immunotherapy Reveals Potential for Innate Beta Cell Regeneration in the Non-Obese Diabetic Mouse Model of Autoimmune Diabetes
Source: PLoS One. 2013 Oct 24;8(10):e78483. doi: 10.1371/journal.pone.0078483 (PMC3813455; doi:10.1371/journal.pone.0078483)

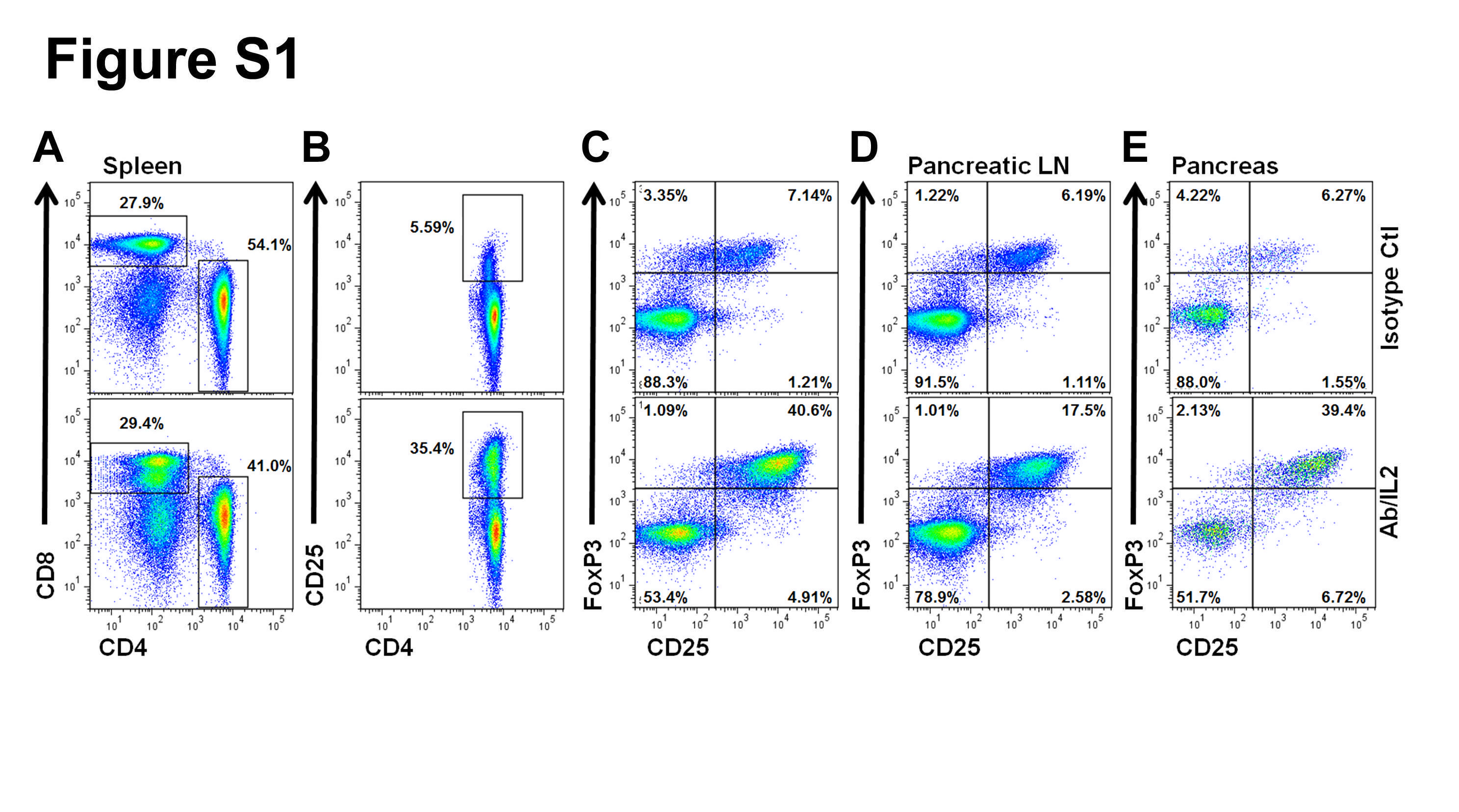

Supplement: Figure S1 — Ab/IL-2 immunotherapy expands Treg cells in recently diabetic NOD mice. Mice recently diagnosed with diabetes were treated for 7 days with Ab/IL-2 or isotype control Ab. Splenocytes, pancreatic lymph node (LN) and pancreatic lymphocytes from n=3 pooled treated mice for each group were analyzed by flow cytometry, as described in methods. Plots shown are gated on CD4+ T cells. Treg cells were selectively expanded by Ab/IL-2 treatment in recently diabetic NOD mice, as previous published (Tang et al [6]) and evidenced here by increased % of CD4+CD25+FoxP3+ cells. (TIF) [file pone.0078483.s001.tif]

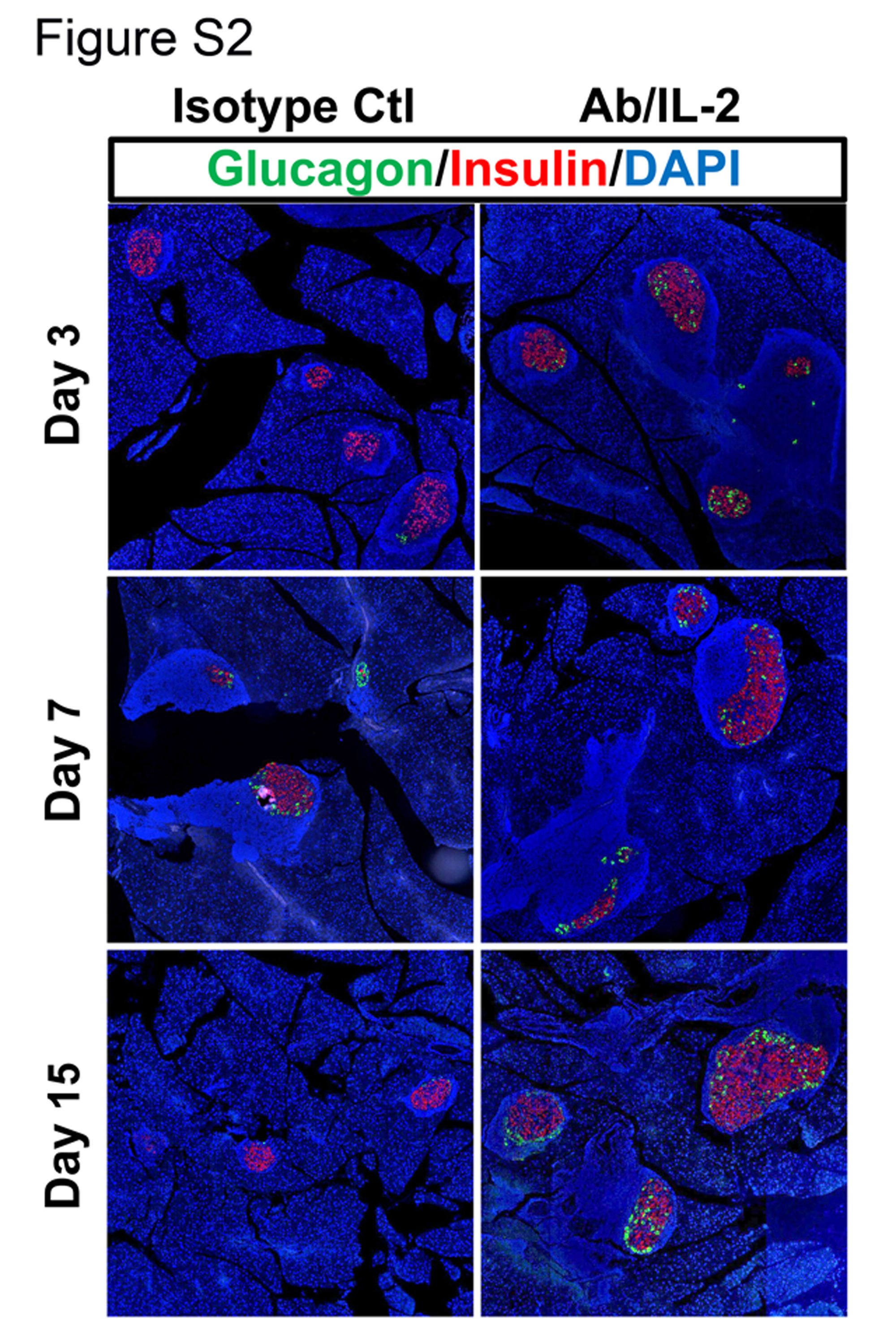

Supplement: Figure S2 — Ab/IL-2 immunotherapy preserves beta cell area. Wide-field images of insulin (red), glucagon (green) and DAPI (blue) stained pancreata presented at 5x magnification. (A, D) At day 3, an equivalent number of islets of comparable size was observed within all pancreata samples. (B, E) By day 7, a progressive loss of islet beta cells was apparent in isotype control treated samples, while Ab/IL-2 samples maintained significant beta cells in islets. (C, F) At day 15, isotype control treated islets were even more reduced in size and number, in comparison to Ab/IL-2 treated samples that had larger islets with more beta cells. (TIF) [file pone.0078483.s002.tif]

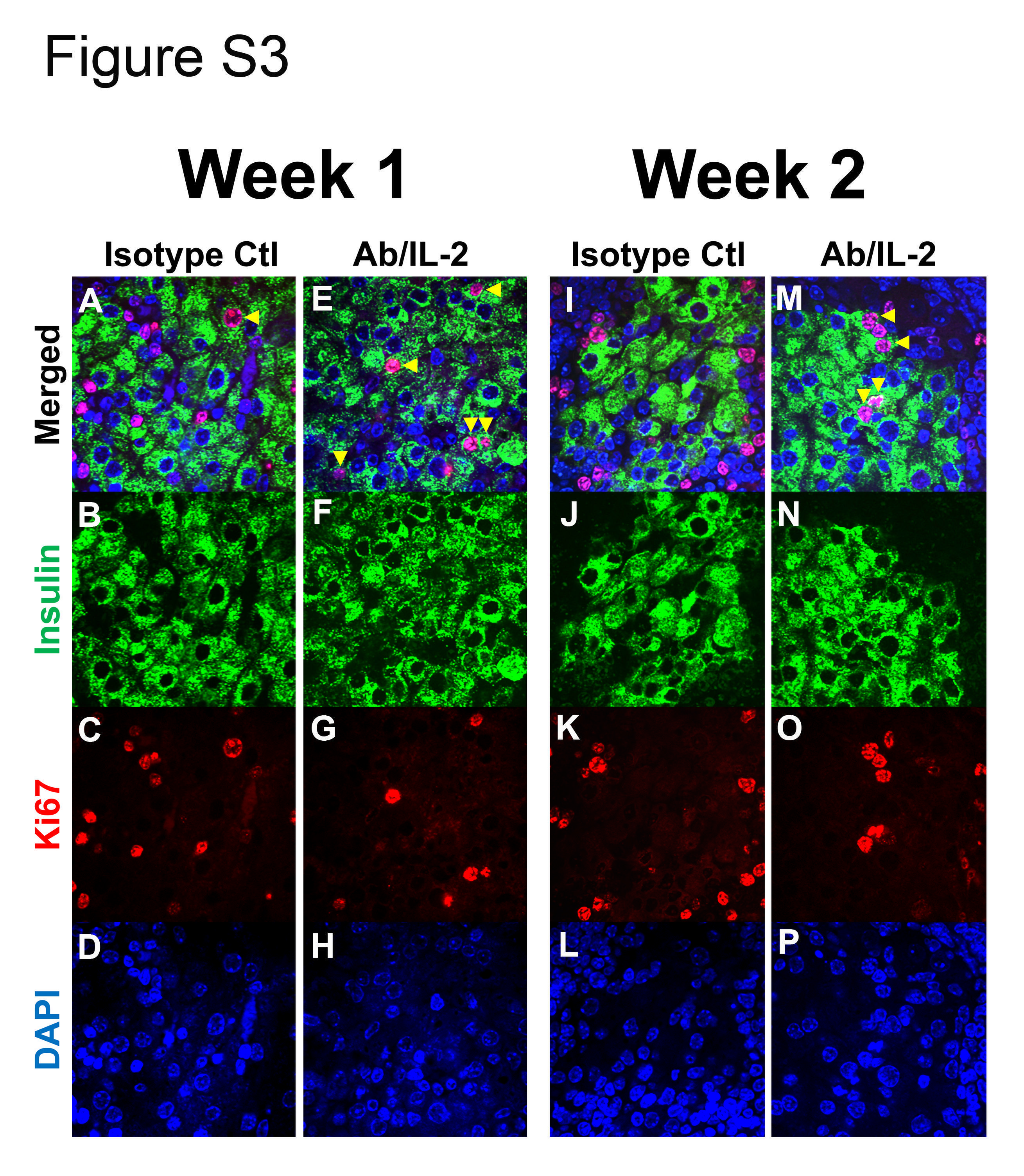

Supplement: Figure S3 — Insulin and Ki67 staining in recently diabetic NOD mice treated with Ab/IL-2 immunotherapy. Single channel and merged images of insulin (green), Ki67 (red) and DAPI (blue) staining in recent onset diabetic NOD mice treated for either one or two weeks with isotype control or Ab/IL-2 immunotherapy. (A-D, and I-L) Isotype control treated samples show few if any proliferating beta cells. (E-H, and M-P) In contrast, Ab/IL-2 treated samples show multiple proliferating beta cells after one or two weeks of treatment. (TIF) [file pone.0078483.s003.tif]

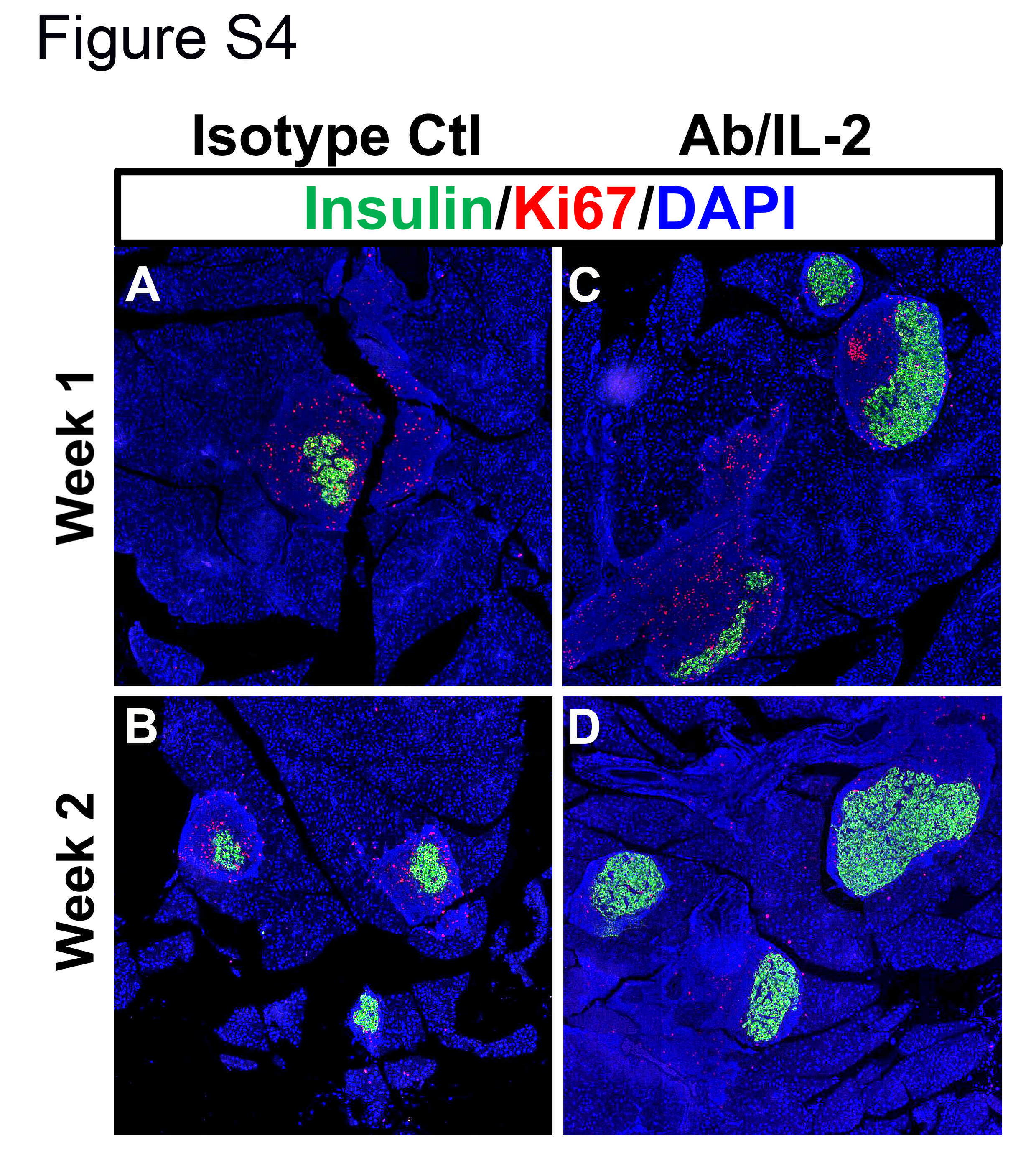

Supplement: Figure S4 — Beta cells demonstrate increased proliferation with Ab/IL-2 immunotherapy. Wide-field images of insulin (green), Ki67 (red) and DAPI (blue) stained pancreata presented at 5x magnification. After one or two weeks of immunotherapy, few Ki67+ beta cells were observed in isotype control treated samples (A, B), while a number of proliferating Ki67+ beta cells were observed in Ab/IL-2 treated samples (C, D). (TIF) [file pone.0078483.s004.tif]

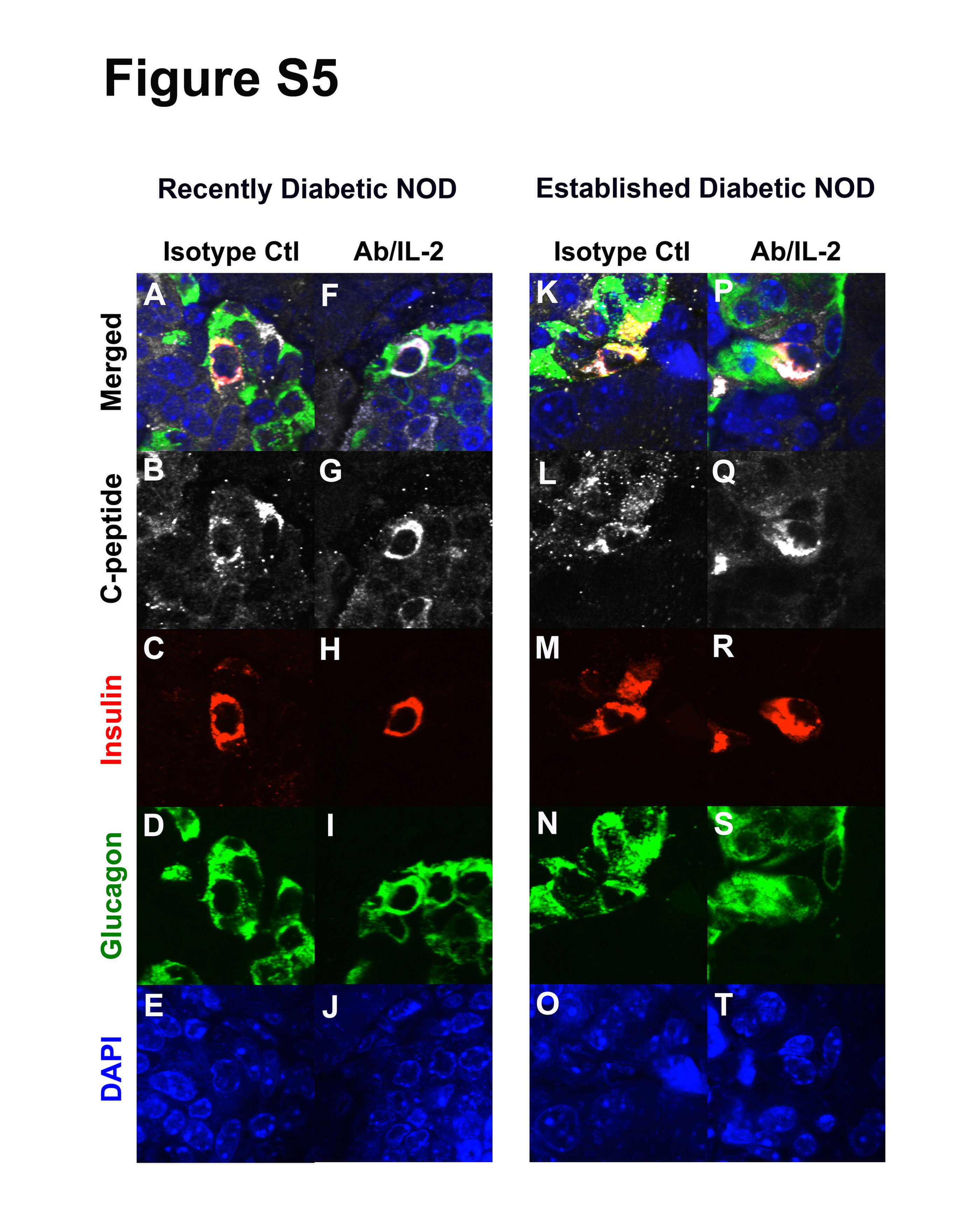

Supplement: Figure S5 — Insulin+/glucagon+ dual-expressing cells co-express C-peptide. Rare insulin (red) and glucagon (green) dual-expressing cells also co-express cytoplasmic C-peptide (white). C-peptide co-expression was observed in recently diabetic NOD mice treated with either isotype control (A-E) or Ab/IL-2 (F-J) immunotherapy, or in established diabetic NOD mice also treated with either isotype control (K-O) or Ab/IL-2 (P-T) immunotherapy. (TIF) [file pone.0078483.s005.tif]

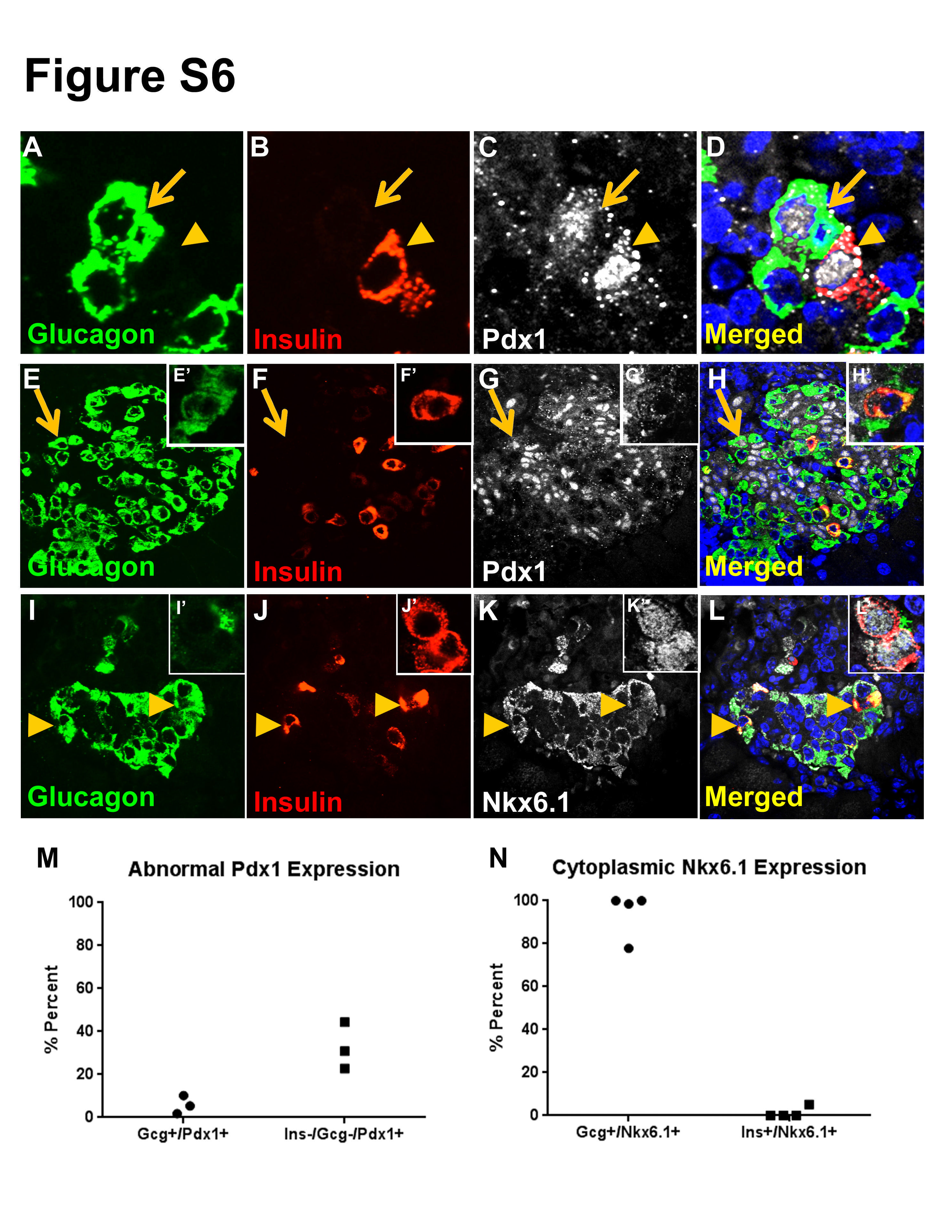

Supplement: Figure S6 — Characterization of abnormal beta cell marker expression in Ab/IL-2 immunotherapy treated islets. Recent-onset NOD mice were treated with Ab/IL-2 or control isotype Ab for 7 days. Pancreata were harvested, processed and stained for insulin (red), glucagon (green), DAPI (blue), and either Pdx1 or Nkx6.1 (white) antibodies. Insulin cells expressed nuclear Pdx1 (arrowhead) as expected (A-D), however occasional glucagon cells showed abnormal expression of nuclear Pdx1 (arrows) (A-H). Widespread hormone negative cells in the center of islets expressed nuclear Pdx1+ and may indicate degranulated beta cells (E-H). Insets show high magnification images of most Pdx1-/insulin+/glucagon+ cells (E’-H’). While insulin+ beta cells normally expressed nuclear Nkx6.1 (I’-L’), most hormone positive cells in diabetic NOD islets showed abnormal cytoplasmic Nkx6.1 expression (I-L), including insulin+/glucagon+ cells (arrowheads). M. Graph representing the percentage of abnormal Pdx1+ expressing cells, including the percent of glucagon+/Pdx1+ alpha cells, and percent of insulin-/glucagon-/Pdx1+ from total islet cell numbers (n=1730 total islet cells, including 943 alpha cells analyzed for Pdx1 expression from n=3 animals.) N. Graph representing the percentage of cells with cytoplasmic Nkx6.1 expression, including the percent of insulin+/Nkx6.1+ beta cells, and percent of glucagon+/Nkx6.1+ alpha cells (n=1514 total islet cells analyzed for cytoplasmic Nkx6.1+ cells, including 816 alpha cells and 158 beta cells from n=4 animals). (TIF) [file pone.0078483.s006.tif]
